# A comparative study on the effects of MRI- and CT-guided interventional therapies on uterine fibroids

**DOI:** 10.12669/pjms.325.10234

**Published:** 2016

**Authors:** Yongxu Mu, Ruiqiang Yan, Junfeng He, Xiaoyan Hu, Qiming Li, Haiyan Liu

**Affiliations:** 1 Yongxu Mu, Department of Intervention, The First Affiliated Hospital of Baotou Medical College, 41 Linyin Road, Kun District, Baotou 014010, China; 2 Ruiqiang Yan, Department of Intervention, The First Affiliated Hospital of Baotou Medical College, 41 Linyin Road, Kun District, Baotou 014010, China; 3 Junfeng He, Department of Intervention, The First Affiliated Hospital of Baotou Medical College, 41 Linyin Road, Kun District, Baotou 014010, China; 4 Xiaoyan Hu, Department of Intervention, The First Affiliated Hospital of Baotou Medical College, 41 Linyin Road, Kun District, Baotou 014010, China; 5 Qiming Li, Department of Intervention, The First Affiliated Hospital of Baotou Medical College, 41 Linyin Road, Kun District, Baotou 014010, China; 6 Haiyan Liu, Department of Intervention, The First Affiliated Hospital of Baotou Medical College, 41 Linyin Road, Kun District, Baotou 014010, China

**Keywords:** MRI, CT, Uterine fibroid, Interventional embolization, Ovary

## Abstract

**Objective:**

To compare the effects of MRI- and CT-guided interventional therapies on uterine fibroids.

**Methods:**

A total of 280 patients with uterine fibroids who were treated in our hospital from August 2008 to February 2014 were selected and divided into a treatment group and a control group by random draw (n=140). The control group and the treatment group were subjected to CT- and MRI-guided interventional therapies for uterine artery embolization.

**Results:**

After three months of treatment, 94.3% and 92.9% of heavy menstrual bleeding and pelvic pressure of the treatment group were relieved respectively, which were similar to those of the control group (92.9% and 92.1% respectively) (P>0.05). The two groups had similar uterine and fibroid sizes before treatment, which were all significantly decreased after treatment (P<0.05) when the treatment group had significantly smaller uteri and fibroids than the control group did (P<0.05). The serum follicle-stimulating hormone, luteinizing hormone, estradiol levels, arterial resistive indices and endometrial thicknesses of the two groups were similar before treatment, which were significantly increased after treatment (P<0.05). Meanwhile, the values of the two groups became significantly different (P<0.05). The treatment group was also significantly less prone to complications such as fever, vaginal bleeding and hematuria than the control group after treatment (P<0.05).

**Conclusion:**

Interventional therapy, especially that guided by MRI, can be performed accurately and safely by mildly affecting the ovary and by promoting the recovery of uterine artery blood flow and endometrial thickness.

## INTRODUCTION

Uterine fibroids (UFs), which are the most common benign tumors of the female reproductive system threatening women of childbearing age, can be classified into intramural fibroids, subserosal fibroids and submucosal fibroids, etc.^1^ Drugs can only mitigate clinical signs temporarily, while surgeries are massively traumatized and thus may not keep the uterus.^2^ Therefore, interventional therapies have been widely applied to treating benign gynecological diseases due to minimal invasion, safety and decrease of complications.^3^ The aim of interventional therapy is to reverse clinical staging by shrinking or eliminating neoplastic foci, to decrease tumor histological grade, and to improve the quality of life.^4^,^5^ Uterine artery embolization (UAE), for example, can relieve or eliminate the clinical symptoms of UFs, recover the menstrual cycle, and reduce uterine and fibroid sizes. Besides, it is of great significance to employ imaging guidance during interventional therapy to prevent injuries to essential organs, thus decreasing complications.^6^,^7^ Being safe, accurate and efficient, computed tomography (CT) guidance directly displays the cross section and reveals the anatomical relationship between lesions and surrounding organs.^8^ Magnetic resonance imaging (MRI), on the other hand, remarkably improves the diagnosis and treatment of gynecological diseases owing to absence of ionizing radiation, multiplanar imaging capability and high contrast resolution.^9^ In this study, we compared the effects of MRI- and CT-guided interventional therapies on uterine fibroids.

## METHODS

### Subjects

A total of 280 patients with uterine fibroids who were treated in our hospital from August 2008 to February 2014 were selected. This study has been approved by the ethic committee of The First Affiliated Hospital of Baotou Medical College, and written consent has been obtained from all patients.

### Inclusion criteria

UFs were diagnosed by clinical, B-scan and pathological examinations; clinically manifested as heavy menstrual bleeding and pelvic pressure; with childbearing history; drugs barely showed effects or UFs relapsed; requirement of preserving the uterus; aged 20-45 years old; married.

### Exclusion criteria

With mental diseases; pregnant women; with primary heart diseases. They were divided into a treatment group and a control group by random draw (n=140). The treatment group was aged 23-44 years old (average: 36.98 ± 5.11), with UFs sized 2-8 cm (average: 4.89 ± 1.09 cm). There were 103 cases of multiple fibroids and 37 cases of single fibroids. There were 89 cases of intermural fibroids, 31 cases of subserosal fibroids and 20 cases of submucosal fibroids. The control group was aged 22-45 years old (average: 36.67 ± 5.09), with UFs sized 2-8 cm (average: 4.81 ± 1.23 cm). There were 98 cases of multiple fibroids and 42 cases of single fibroids. There were 91 cases of intermural fibroids, 29 cases of subserosal fibroids and 20 cases of submucosal fibroids. The age, fibroid size, as well as multiplicity and type of fibroids of the two groups were similar (P>0.05).

### Treatment methods

All patients were subjected to UAE by using an emulsion of embolic agent lipiodol and pingyangmycin, with Ultravist 370 as the contrast agent. All patients, in the supine position, were monitored, locally anesthetized by 5% lidocaine, and catheterized through the right femoral artery. Under CT guidance for the control group and MRI guidance for the treatment group, contralateral iliac arteries were selectively imaged by 4F cobra catheters, and 3-5 cm deep inside the uterine artery was imaged by ultraselection when UAE was performed by ipsilateral uterine artery angiography. The puncture site was pressed for 15 minutes and then compression-bandaged for six hour while maintaining the supine position for 24 hour. During imaging, contrast agent was injected at the speed of 6 ml/s (20 ml in total), and UAE was conducted by using sodium alginate microspheres (diameter: 700-900 μm) comprising antibiotics and contrast agent. After surgeries, antibiotics were given, and blood routine examination and liver function test were carried out.

By using GE Light-speed 32-slice helical CT, the position of fibroid was scanned (layer thickness: 5 mm, layer distance: 5 mm) to select the optimum layer as well as to determine its linear distance from the edge of foci and the maximum depth, angle of the needle. MRI was performed by employing Philips 0.23 T open resistive MRI system with fast spin echo sequence. Parameters: Repetition time, 125 ms; echo time, 7.0 ms; field of view, 300 mm; matrix, 270×270; time, 24 s. During application, MRI multifunctional coil was fixed near the site awaiting puncture to which the puncture needle was targeted to obtain the scan image and to display lesions and surrounding structures. Puncture sites and route were determined based on the image and signal-intensified lesions.

### Observation indices

Heavy menstrual bleeding and pelvic pressure were observed. By using PHILIPS iU22 color Doppler ultrasound scanner, uterine and fibroid sizes were measured three months before and after treatment, with the probe longitudinally detecting the length and depth and then horizontally detecting the transverse diameter.

On the third days of the menstrual cycles three months before and after treatment, fasting venous blood (3 ml) was collected at 8:00 am, centrifuged and stored in a -20ºC refrigerator prior to use. Serum follicle-stimulating hormone (FSH), luteinizing hormone (LH) and estradiol (E2) levels were detected by the chemiluminescence enzyme immunoassay with an automated chemistry immunoassay system (Beckman Coulter, Inc., USA) according to the kit’s instructions. Postoperative complications such as abdominal pain, fever, vaginal bleeding and hematuria were observed.

Endometrial thicknesses and uterine artery blood flow parameters were determined with PHILIPS iU22 color Doppler ultrasound scanner three months before and after treatment.

### Statistical analysis

All data were analyzed by SPSS 17.0. The numerical data were expressed as mean ± standard deviation and compared by t test and Chi-square test. The categorical data were compared by Chi-square test. P<0.05 was considered statistically significant.

## RESULTS

### Improvement of clinical symptoms

After three months of treatment, 94.3% and 92.9% of heavy menstrual bleeding and pelvic pressure of the treatment group were relieved respectively, which were similar to those of the control group (92.9% and 92.1% respectively) (P>0.05) (Table-I).

**Table-I:** Improvement of clinical symptoms (n).

*Group*	*Case No.*	*Heavy menstrual bleeding*	*Pelvic pressure*
Treatment group	140	132 (94.3%)	130 (92.9%)
Control group	140	130 (92.9%)	129 (92.1%)
χ^2^		0.068	0.089
P		>0.05	>0.05

### Changes of uterine and fibroid sizes

The two groups had similar uterine and fibroid sizes before treatment, which were all significantly decreased after treatment (P<0.05) when the treatment group had significantly smaller uteri and fibroids than the control group did (P<0.05) (Table-II).

**Table-II:** Changes of uterine and fibroid sizes.

*Group*	*Case number (n)*	*Uterine size*		*Fibroid size*	

		*Before*	*After*	*Before*	*After*
Treatment group	140	145.38±10.37	66.38±9.22	26.93±4.93	6.11±1.73
Control group	140	144.98±11.63	95.29±10.38	26.98±5.13	7.98±2.03
t		0.239	13.746	0.078	6.003
P		>0.05	<0.05	>0.05	<0.05

### Changes of sex hormone levels

The serum FSH, LH and E2 levels of the two groups were similar before treatment, which were significantly increased after treatment (P<0.05). Meanwhile, the values of the two groups became significantly different (P<0.05) (Table-III).

**Table-III:** Changes of sex hormone levels (x ± s).

*Group*	*Case number (n)*	*FSH (mIU/ml)*		*LH (mIU/ml)*		*E2 (pg/ml)*	

		*Before*	*After*	*Before*	*After*	*Before*	*After*
Treatment group	140	7.28 ± 1.76	9.49 ± 2.19	4.38 ± 2.09	5.13 ± 2.78	78.83 ± 10.34	88.38 ± 13.42
Control group	140	7.29 ± 1.63	14.38 ± 1.67	4.39 ± 1.68	7.67 ± 2.01	78.48 ± 8.33	99.78 ± 13.24
t		0.067	8.552	0.018	7.399	0.399	4.092
P		>0.05	<0.05	>0.05	<0.05	>0.05	<0.05

### Changes of uterine artery blood flow parameters and endometrial thickness

The arterial resistive indices (RIs) and endometrial thicknesses of the two groups were significantly elevated after treatment (P<0.05). In the meantime, the values of the two groups were significantly different (P<0.05) (Table-IV).

**Table-IV:** Changes of uterine artery blood flow parameters and endometrial thickness (x ± s).

*Group*	*Case number (n)*	*RI*		*Endometrial thickness (mm)*	

		*Before*	*After*	*Before*	*After*
Treatment group	140	0.77 ± 0.16	0.82 ± 0.22	6.08 ± 1.78	9.73 ± 3.02
Control group	140	0.78 ± 0.18	0.93 ± 0.29	6.09 ± 1.43	12.82 ± 2.09
t		0.056	6.378	0.034	7.446
P		>0.05	<0.05	>0.05	<0.05

### Complications

The treatment group was significantly less prone to complications such as fever, vaginal bleeding and hematuria than the control group after treatment (P<0.05) (Table-V).

**Table-V:** Complications after treatment (n).

*Group*	*Case number (n)*	*Abdominal pain*	*Fever*	*Vaginal bleeding*	*Hematuria*
Treatment group	140	4 (2.9%)	3 (2.1%)	6 (4.3%)	3 (2.1%)
Control group	140	5 (3.6%)	11 (7.9%)	19 (13.6%)	12 (8.6%)
t		0.078	5.399	4.092	7.898
P		>0.05	<0.05	<0.05	<0.05

## DISCUSSION

As a common disease in clinical practice, UF contains a rich vascular network. Anatomically speaking, there are both inner and outer vascular networks. The former refers to originates from the outer network and exists inside UFs, providing blood for UFs. The latter, which originates from original branches of the uterine artery, is located in the pseudocapsule of UF surface.^10^,^11^

For UF treatment, UAE is minimally invasive, facile and allows quick recovery, without causing blood loss or requiring hysterectomy. Particularly, keeping the uterus intact is more ethical and can promote the physical and psychological recoveries. Meanwhile, UFs undergo ischemia, necrosis and atrophy after UAE before being removed.^12^ UFs are benign smooth-muscle tumors, with blood supplied by bilateral uterine arteries, so UAE can immediately mitigate heavy menstrual bleeding and then pelvic pressure by decreasing blood flow in the uterus.^13^ After three months of treatment, 94.3% and 92.9% of heavy menstrual bleeding and pelvic pressure of the treatment group were relieved respectively, which were similar to those of the control group (92.9% and 92.1% respectively) (P>0.05).

Color Doppler ultrasound scanning is able to disclose the dilation and distortion of the uterine artery and peritumoral arterial branches, vascular dysplasia, as well as abnormally rich intratumoral and peritumoral blood flows.^14^ By reducing blood flow, UAE only induces UF necrosis other than massive necrosis of the uterus, mainly because UF cells cannot endure ischemia and hypoxia as effectively as normal uterine smooth muscle cells do owing to active division.^15^ In the meantime, the uterus escapes from massive necrosis due to blood supply from abundant pelvic collateral circulation and vascular network, together with the traffic branch of iliolumbar artery, ovarian artery, vaginal artery and sacral artery. However, early pathological changes of UFs cannot be detected accurately based only on clinical signs and imaging characteristics. Since MRI gives better image quality than CT does by displaying UF foci more clearly without being affected by bones, MRI is preferred to CT now in order to ensure successful UAE.^16^ In this study, the two groups had similar uterine and fibroid sizes before treatment, which were all significantly reduced after treatment (P<0.05) when the treatment group had significantly smaller uteri and fibroids than the control group did (P<0.05), suggesting that the patients were treated more effectively by MRI-guided UAE.

As a female gonad, the ovary is mainly responsible for ovulation and secretion of female hormones. Upon oversecretion, however, less FSH and LH are secreted by the pituitary gland through negative feedback, so ovarian functions can be indirectly evaluated by measuring the levels of related hormones.^17^ Ovarian dysfunction is a severe complication of conventional interventional therapies, particularly for the women of childbearing requirements. Inappropriate interventional therapies may weaken fertility by injuring ovarian functions. In addition, the patients who are treated with lipiodol or small-sized embolic agent particles and the over-embolized ones are subject to partial or complete necrosis of the ovary, thus suffering severe prognosis.^18^ Therefore, the methods and agents for embolization should be carefully selected prior to treatment to avoid unnecessary damages. Moreover, in some cases, UAE still fails to cease blood flow in the uterine artery, and embolic agents may enter the ovary and affect blood supply therein accordingly. To this end, MRI guidance is employed to shun ovarian branch of the uterine artery and to clearly display foci and the surrounding structures, thereby allowing real-time image feedback and non-axial entrance, monitoring the locations of related tissues in random directions, and minimizing injuries to normal tissues.^19^ In this study, the serum FSH, LH and E2 levels of the two groups were similar before treatment, which were significantly raised after treatment (P<0.05). Meanwhile, the values of the treatment group significantly exceeded those of the control group (P<0.05).

The sequence of interventional MRI is different from that of diagnostic MRI due to the lower requirement of tissue image resolution. During embolization, pelvic arteries intertwine when anterior and posterior internal iliac arteries of different individuals are imaged. To circumvent thrombosis-induced complications because of intimal injury, MRI is used to determine the origin of uterine artery and to facilitate the recovery of uterine artery blood flow parameters and endometrial thickness. The RIs and endometrial thicknesses of both groups were significantly augmented after treatment (P<0.05). In the meantime, the values of the two groups differed significantly (P<0.05).

Generally, UAE barely results in complications, but that guided by CT gives contrary outcomes owing to low accuracy.^20^ Open MRI guidance, as a simple, frameless stereotactic technique, can prevent possible shift induced by ordinary stereotactic techniques as well as the deviations caused by tissue movement. Furthermore, it manages to minimize injuries to normal tissues and structures around lesions, which thus ensures safe diagnosis and treatment and decreases the odds of complications.^21^ After treatment in this study, the treatment group was significantly less prone to complications such as fever, vaginal bleeding and hematuria than the control group (P<0.05).

In summary, interventional therapy, especially that guided by MRI, can be conducted accurately and safely by slightly affecting the ovary and by facilitating the recovery of uterine artery blood flow and endometrial thickness. Hence, this method is worthy of wide application in clinical practice.
